# Ten Years of Extracellular Matrix Proteomics: Accomplishments, Challenges, and Future Perspectives

**DOI:** 10.1016/j.mcpro.2023.100528

**Published:** 2023-03-12

**Authors:** Alexandra Naba

**Affiliations:** 1 Department of Physiology and Biophysics, University of Illinois at Chicago, Chicago, Illinois, USA; 2 University of Illinois Cancer Center, Chicago, Illinois, USA

**Keywords:** matrisome, microenvironment, protein solubility, posttranslational modifications, biomarkers

## Abstract

The extracellular matrix (ECM) is a complex assembly of hundreds of proteins forming the architectural scaffold of multicellular organisms. In addition to its structural role, the ECM conveys signals orchestrating cellular phenotypes. Alterations of ECM composition, abundance, structure, or mechanics have been linked to diseases and disorders affecting all physiological systems, including fibrosis and cancer. Deciphering the protein composition of the ECM and how it changes in pathophysiological contexts is thus the first step toward understanding the roles of the ECM in health and disease and toward the development of therapeutic strategies to correct disease-causing ECM alterations. Potentially, the ECM also represents a vast, yet untapped reservoir of disease biomarkers. ECM proteins are characterized by unique biochemical properties that have hindered their study: they are large, heavily and uniquely posttranslationally modified, and highly insoluble. Overcoming these challenges, we and others have devised mass-spectrometry–based proteomic approaches to define the ECM composition, or “matrisome,” of tissues. This first part of this review provides a historical overview of ECM proteomics research and presents the latest advances that now allow the profiling of the ECM of healthy and diseased tissues. The second part highlights recent examples illustrating how ECM proteomics has emerged as a powerful discovery pipeline to identify prognostic cancer biomarkers. The third part discusses remaining challenges limiting our ability to translate findings to clinical application and proposes approaches to overcome them. Lastly, the review introduces readers to resources available to facilitate the interpretation of ECM proteomics datasets. The ECM was once thought to be impenetrable. Mass spectrometry–based proteomics has proven to be a powerful tool to decode the ECM. In light of the progress made over the past decade, there are reasons to believe that the in-depth exploration of the matrisome is within reach and that we may soon witness the first translational application of ECM proteomics.

## Highlights

• ECM alterations cause or accompany diseases and disorders of all organ systems.
• Proteomics is a method of choice to profile the composition of the ECM of tissues.
• ECM proteomics can identify novel prognostic and diagnostic biomarkers.
• ECM proteomics can uncover proteins playing functional roles in disease etiology.
• Further technical advances are needed to capture the diversity of ECM proteoforms

## Graphical Abstract

**Figure:**
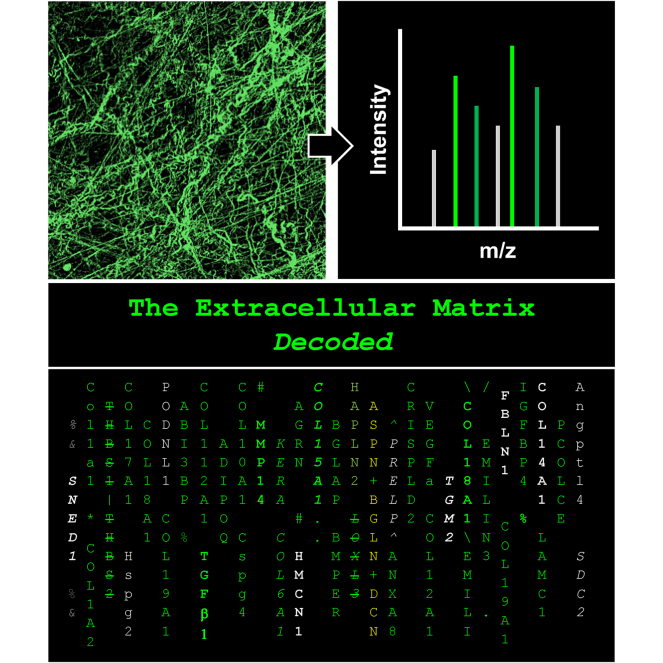

## In Brief

The extracellular matrix (ECM) is a complex assembly of hundreds of proteins forming the architectural organizer of multicellular organisms. Over the past decade, bottom-up proteomics has become the method of choice to profile the composition of the ECM. This article reviews the state of the art in ECM proteomics research and illustrates how ECM proteomics has emerged as a powerful discovery pipeline for cancer biomarkers. This review also introduces resources available to facilitate the study of the ECM using proteomics.

## The Extracellular Matrix: the Master Organizer of Multicellular Organisms

The extracellular matrix (ECM) is a complex assembly of proteins forming the architectural scaffold of all multicellular organisms (1, 2, 3). As such, the ECM guides cell polarization and serves as a substrate to cell migration, it organizes cells into tissues and tissues into organs, and confers mechanical properties to tissues. In addition to its structural roles, the ECM exerts signaling functions through mechanotransduction (4, 5). It also provides biochemical cues interpreted by cells *via* cell-surface receptors (*e.g.*, integrins (6), syndecans, adhesion GPCRs (7)) that orchestrate most, if not all, cellular functions, from cell proliferation and survival to adhesion and migration, to stemness and differentiation. The ECM thus plays critical roles during development, growth, and other physiological processes including wound healing and aging (8, 9, 10, 11, 12). Simply put, the ECM is essential for multicellular life.

## The Extracellular Matrix: a key Player in Disease Etiology and Progression

The ECM is a dynamic compartment that undergoes compositional turnover and structural remodeling mediated by both enzymatic and nonenzymatic processes. Disruption of ECM homeostasis, caused by mutations in ECM genes (13), by an imbalance between ECM production and degradation, or by inadequate ECM remodeling, results in disorders and diseases affecting all physiological systems (14, 15, 16) including the musculoskeletal system (*e.g.*, Ehlers–Danlos syndrome (17), arthritis), the skin (*e.g.*, scleroderma (18), epidermolysis bullosa (19)), the cardiovascular system (*e.g.*, Marfan syndrome (20)), the respiratory system (lung fibrosis (21)), and the excretory system (*e.g.*, Alport syndrome, Goodpasture syndrome, renal fibrosis (22, 23)), to list a few. In addition, excessive ECM accumulation is a hallmark of fibrosis (24) and cancer (25, 26, 27). The extent of ECM deposition in the context of cancer, assessed by the tumor:stroma ratio, has been shown to be of prognostic value for patients with colorectal cancer (28, 29). Nine genes of the 70-gene MammaPrint panel used for early breast cancer diagnosis (30) are ECM genes. ECM proteins present the advantage of being readily accessible, outside the cells. Consequently, they can be used for the targeted delivery of imaging agents (31, 32, 33) or drugs, for example, by using bispecific agents composed of a moiety recognizing a disease-specific ECM protein and an immunomodulatory cytokine (34, 35, 36). Lastly, it has been proposed that modulating the architecture or biophysical properties of the ECM or ECM–cell interactions could be a valid therapeutic approach in various contexts (37, 38, 39, 40, 41, 42). The ECM thus constitutes a large reservoir of biomarkers and potential therapeutic targets. Yet, while some proteins (*e.g.*, fibronectin, elastin) or families of proteins (*e.g.*, collagens, tenascins) of the ECM have been extensively studied, the ECM as a whole, remained, until recently, largely underexplored (43) and uncharted (44).

## Challenges Posed by ECM Proteins

The very biochemical properties allowing ECM proteins to assemble into an architectural scaffold capable of withstanding significant mechanical stress and deformations have hindered our ability to study the global composition of the ECM. The core, structural proteins of the ECM tend to be very large, on average 1045 amino acids long. ECM proteins undergo extensive intracellular and extracellular posttranslational modifications (PTMs), including glycosylation, lysine and proline hydroxylation for collagens and collagen-domain-containing proteins that contribute to the stabilization of the triple-helical structure of collagens (45), and glycation. ECM proteins also assemble into higher-order molecular structures established *via* hydrogen bonds *(e.g.*, collagen triple-helical structures (46, 47)), disulfide bonds (*e.g.*, fibronectin dimers (48)), and covalent cross-links (*e.g.*, elastin (49), collagens (50)). These biochemical properties contribute to making ECM proteins highly insoluble and, hence, challenging to study using standard biochemical approaches like SDS-PAGE, immunoprecipitation and pull-down assays or mass spectrometry (MS). Because of their high insolubility, ECM proteins are underrepresented in global proteomic datasets. Further contributing to this underrepresentation is the fact that, apart from a few exceptions, the ECM represents a small fraction of healthy organ and tissue mass.

The second challenge limiting the comprehensive characterization of the ECM is its broad dynamic range in terms of protein abundance. The ECM is comprised of very large and highly abundant structural ECM components, which can generate many peptides (for example, there are 121 trypsin cleavage sites in the alpha 1 chain of collagen I), and also smaller secreted factors, such as ECM-remodeling enzymes, growth factors, or morphogens, present in much lower abundance. This limitation is not unique to the ECM, and advances in instrumentations and methods to fractionate protein and peptide samples, that will not be discussed here, have been key to capture the complexity of different subproteomes and are now being applied to ECM proteomics (see below).

The first attempts at profiling the protein composition of the ECM of ECM-rich tissues, like the cartilage, or following ECM enrichment for other tissues, employed SDS-PAGE or 2D gel electrophoresis to separate the subsets of ECM proteins that could be solubilized, followed by liquid chromatography coupled to tandem mass spectrometry (LC-MS/MS). These studies reported the detection of up to a few dozen structural ECM proteins. At the time, this was no small feat and these early studies have been instrumental in helping shape the field of ECM proteomics (51, 52, 53, 54, 55, 56). Of note, with sample preparation protocols tailored to account for the unique challenges posed by ECM protein (insolubility, extensive glycosylation), separation by 1D SDS-PAGE resulted in the identification of nearly 100 distinct extracellular proteins (57, 58).

However, for most studies, known ECM proteins, expected to be detected in those tissues, were not identified. One may then ask: how can we ensure capturing potential novel ECM proteins or proteins not known to be present in these tissues? And indeed, the third challenge our field faced when attempting to characterize, in an unbiased manner, the protein composition of the ECM of tissues, was the lack of a defined parts list to systematically annotate experimental output. As a result, in the early days of ECM proteomics, many proteins listed as “ECM” proteins were in fact intracellular proteins involved in cell–ECM adhesions or secreted proteins but not incorporated in the ECM. Conversely, proteins for which no prior knowledge existed would fail to be annotated as belonging to the ECM. This represented a significant limitation to any attempt aiming to identify biomarkers of diseased states. It thus became obvious that tailored experimental and analytical approaches would be needed to decipher the complexity of the ECM.

This review will discuss the latest developments in ECM proteomics, from enhancement in sample preparation and analytical methods to the application of ECM proteomics for the purpose of biomarker and therapeutic target discovery with a focus on cancer. As part of the Special Issue on Clinical Proteomics, this article will highlight selected studies performed on clinical samples or rodent models of human diseases that show translational promise. Of note, ECM proteomics is also applied to study the ECM of many multicellular organisms, for example, zebrafish (59, 60, 61), drosophila (62), or planarians (63) or to the ECM produced by cells in culture. These studies are instrumental to advance fundamental knowledge of the ECM in the context of development, health, and disease. In addition, this review will focus on bottom-up MS-based proteomics, but, it is worth noting that other MS-based modalities can be employed to study different facets of the ECM, including the glycosylation patterns of ECM proteins using glycomics (64, 65, 66, 67), the identification of cleavage fragments of ECM proteins using degradomics (68), or the localization and distribution of ECM proteins using imaging MS (69, 70).

## Defining the “Matrisome” and Establishing a Framework for ECM Proteomics Research

In 2012, we published in this journal an article describing a two-pronged approach to define the protein composition of the ECM of tissues (71). While prior studies had attempted to overcome some of the limitations described above (for example, by decellularizing samples or extracting ECM proteins in guanidine hydrochloride), we set up to tackle them all. In brief, we first took advantage of the differential solubility of intracellular and ECM proteins to deplete non-ECM proteins by sequential incubations in extraction, or decellularization, buffers concomitantly enriching for ECM proteins. Observing that incubation in 8 M urea and 100 mM DTT did not fully solubilize ECM-enriched samples and suspecting that many ECM components would be found in the insoluble material, we processed “crude” 8 M-urea-resuspended samples. We then hypothesized that deglycosylating ECM proteins would enhance trypsin accessibility and thus treated samples with Peptide-N-glycosidase F (PNGaseF). We further preincubated deglycosylated ECM-enriched protein suspension with LysC, a smaller protease capable of digesting tightly folded proteins, prior to tryptic digestion. To capture a broad range of ECM components, we fractionated peptide samples using off-gel electrophoresis. Last, to ensure the correct identification and quantification of all ECM proteins, we stipulated the ECM-specific PTMs lysine and proline hydroxylations as variable modifications for database search. Indeed, proline represents 19% of the amino acid sequence of the alpha 1 chain of collagen I and is found in positions X and Y of X-Y-Gly repeats and is often hydroxylated (45).

In parallel, we developed a robust nomenclature to annotate and classify ECM proteins. In brief, we used sequence analysis and the characteristic domain-based organization of ECM proteins (72, 73) to derive an ECM parts list (71, 74, 75, 76) that we called the “matrisome.” This term was originally coined by Dr George Martin, in the early 1980s, to describe the components of a specialized type of ECM called the basement membrane (77). In 2012, we proposed to expand the definition of the “matrisome” to describe the compendium of genes predicted to encode structural ECM proteins, *i.e.*, the “core matrisome” including collagens, noncollagen ECM glycoproteins, and proteoglycans. The “matrisome” also included proteins not directly contributing to the structure of the ECM meshwork but are involved in ECM homeostasis and signaling functions, such as ECM remodeling enzymes and growth factors capable of interacting with ECM proteins (71, 74, 75, 76).

The combination of an experimental approach, tailored to study ECM proteins, and the definition of the matrisome parts list resulted in the characterization of the ECM of murine lung and colon samples. These comprised well over 100 proteins, including both core matrisome and matrisome-associated proteins, such as the ECM-cross-linking enzymes from the lysyl oxidase and transglutaminase families, ECM-degrading enzymes of the matrix metalloproteinases family, growth factors, and interleukins (71).

A broad distribution *via* an open access publication (71) and a companion website (http://matrisome.org), easy-to-follow step-by-step protocols and videos (78, 79), and the utilization of either commercially available reagents or reagents easy to prepare in any wet-lab with basic biochemistry knowledge have allowed our methods to become broadly adopted and built upon.

## Overview of Current ECM Proteomic Workflows

**Fig. 1:**
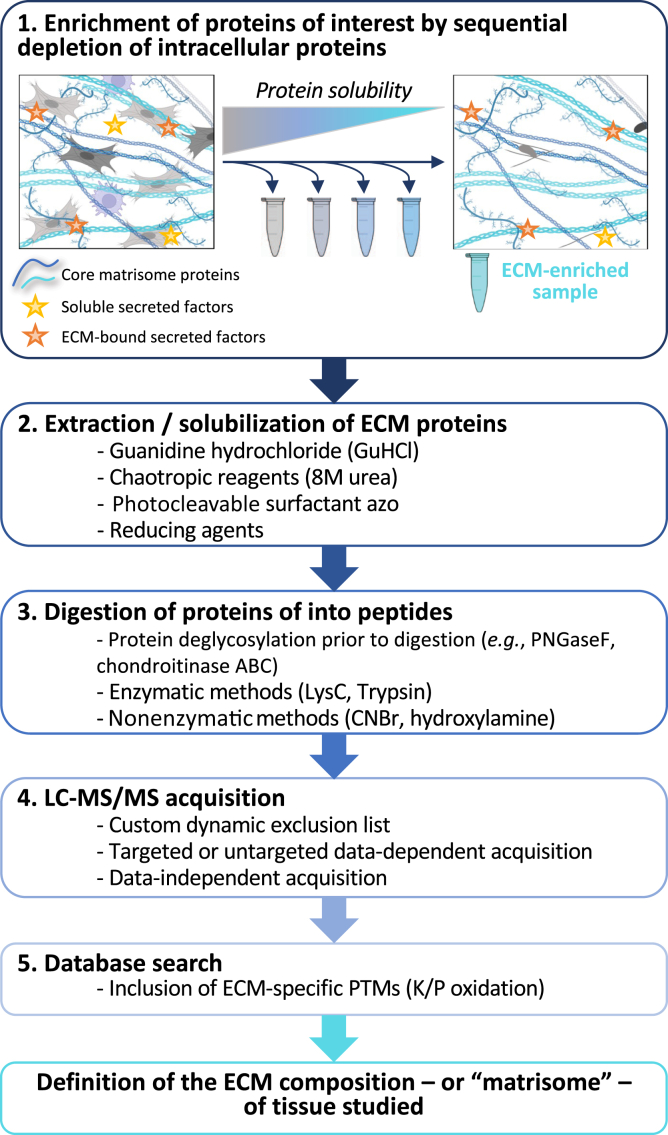
**Overview of ECM proteomics workflows**. Current ECM proteomics workflows can be divided into 5 broad steps: 1) ECM protein enrichment, 2) protein solubilization, 3) protein digestion into peptides, 4) LC-MS/MS acquisition, and 5) database search. Schematics presented in panel 1 are adapted from the template “A Closer Look Into the Extracellular Matrix (ECM) Proteome” by BioRender.com (2022). ECM, extracellular matrix.

Over the past decade, bottom-up proteomics has become the method of choice to profile the protein composition of the ECM. While nuances exist in the ECM-enrichment/decellularization protocols employed, in the reagents used to extract or solubilize ECM proteins or in the enzymes used for ECM protein deglycosylation or to generate peptides, all current workflows are based on a similar stepwise process illustrated in Figure 1. Recent developments are briefly reviewed here.

### Sample Preparation Methods

Similar to our pipeline, the Schiller and Mann laboratories have devised an ECM-enrichment strategy using detergent that generates four fractions, three containing proteins of low to intermediate solubility and an insoluble ECM-enriched fraction (80). They further proposed to analyze not only the insoluble fraction but also those of intermediate solubilities by LC-MS/MS to derive a quantitative detergent solubility profile (QDSP) of the ECM of the murine lung (80), human fibrotic lung and skin (81), aging human lung (82), and, more recently, the different regions of the murine brain (83). Upon collation of lists of matrisome proteins detected in each fraction, the QDSP protocol is one of the protocols that results in the largest number of matrisome proteins detected. However, it is a costly procedure, since it requires running a larger number of samples. In addition, it is important to appreciate that the detection of matrisome proteins in fractions of low or intermediate solubility can correspond to the pool of intracellular matrisome proteins not yet secreted or to matrisome proteins found in the extracellular space but not as tightly assembled into the insoluble ECM meshwork. Yet, QDSP is a powerful approach, for researchers interested in evaluating changes in matrisome protein solubility in different physiological or pathological states.

The Hansen laboratory largely contributed to develop approaches for ECM proteomics, including the use of nonenzymatic approaches to digest proteins using hydroxylamine or cyanogen bromide (84, 85). More recently McCabe, Hansen, and colleagues conducted an extensive comparative study and assessed the efficiency of five different ECM enrichment methods, four protein extraction methods, and two deglycosylation methods to profile the ECM of several murine tissues (86). However, this study only focused on the impact of experimental parameters on the identification of core matrisome proteins, only partially capturing the complexity of the ECM. Other recent comparative studies of protocols, differing in the methods employed for ECM enrichment, ECM protein solubilization, and/or peptide generation, have revealed that there is a large overlap in the matrisome proteins identified, and that there are also, as expected, subsets of proteins uniquely identified using one protocol or another (86, 87, 88).

The Amorim lab has proposed a simpler two-step fractionation approach using commercially available MS-compatible reagents and separating tissue lysates into two pools of protein: detergent soluble and detergent insoluble (89). Similarly, the Ge lab has developed a photocleavable detergent called azo and applied it in a two-step fractionation approach to characterize the ECM composition of murine mammary tumors (90). As anticipated, in both studies, the pool of soluble proteins contained a larger number of matrisome-associated proteins and the analysis of the pool of insoluble proteins contained a larger number of core matrisome proteins. As discussed above, the detection of matrisome proteins in fractions of low or intermediate solubility can correspond to intracellular matrisome proteins or to matrisome proteins found in the extracellular space but not incorporated in the insoluble ECM meshwork. In addition, the initial *in silico* prediction of the matrisome-associated genes was intentionally broad and included all members of protein families even if only one member of that family was known to act extracellularly (*e.g.*, cathepsins, cystatins, or annexins). Orthogonal validation, for example, using tissue staining, is thus necessary to gain information on protein localization, which, in turn, will provide crucial information on protein function.

ECM protein deglycosylation plays a key role in enhancing trypsin accessibility. Common protocols employ PNGaseF, an enzyme that cleaves N-linked glycans. However, some peptides may be resistant to PNGase F (91), and PNGaseF will not cleave O-linked glycans. Removal of O-linked glycans can be achieved by exoglycosidases (*e.g.*, sialidase, O-glycosidase, galactosidase). Recent protocols have reported other deglycosylation methods using trifluoromethanesulphonic acid or enzymes to cleave the glycosaminoglycan (GAG) moieties of proteoglycans such as chondroitinase ABC, heparin lyase, heparanase, and keratanase. While deglycosylation may enhance the release of peptides and thus protein discovery and quantification, it is worth noting that not capturing the glycosylation profile of ECM components only results in a partial view of this compartment (see below).

In conclusion, there is no “best” or no “right” sample preparation method. Different methods will yield different results. It is thus important to consider the strengths and limitations of each protocol available, as well as more practical considerations like ease of use, reagent availability, time, and cost. Ultimately, researchers should opt for the method that is best suited to investigate the one facet of the multifaceted ECM they are interested in, for example, identifying the largest number of proteins, focusing on the more insoluble components of the ECM to understand the mechanisms of assembly of the ECM meshwork, or detecting ECM remodeling enzymes present in differential abundance between healthy and diseased tissue for the purpose of drug target discovery.

### Tailoring Data Acquisition and Database Search to Enhance Matrisome Protein Discovery

Over the past decade, ECM proteomics has adopted methods broadly used for global proteomics such as tandem-mass labels (TMT, iTRAQ) for accurate determination of protein abundance or the inclusion of peptide fractionation using high-pH reversed-phase liquid chromatography (79). While data-dependent acquisition remains the main MS modality for ECM profiling, a few recent studies have also employed data-independent acquisition (92, 93, 94, 95).

For accurate protein identification and estimation of protein abundance, it is imperative to maximize peptide-to-spectrum matching, which requires prior knowledge, in particular on the nature of the posttranslational modifications that can affect proteins of interest. For collagens and collagen-domain-containing proteins, these include oxidation (or hydroxylation) of lysines and prolines. These variable modifications are typically not selected by default for database searches, since they increase the search time for global proteomic studies and have limited impact on the output. However, we and others have shown that omitting lysine and proline oxidation results in the underestimation of the number and abundance of matrisome proteins present in samples (79, 96).

To overcome the limitation posed by the large dynamic range of matrisome protein abundance, we attempted to implement a custom dynamic exclusion approach to “ignore” all peptides derived from the most abundant proteins detected in our samples (97). To do so, we constructed a list of 2332 unique peptides including all identified peptides from the alpha 1 and alpha 2 chains of collagen I (Col1a1 and Col1a2), the alpha 1 chain of collagen type III (Col3a1), and the alpha 2 chain of collagen IV (Col4a2). We also excluded all identified peptides from vimentin, myosin-9, and filamin-A, three nonmatrisome proteins detected with high spectral count in our samples. This attempt had only modest success in increasing the number of matrisome proteins identified; however, it resulted in a significant increase in the number of matrisome peptide spectrum matches and, consequently, led to a more accurate quantification of the proteins detected across our experimental conditions (97). A drawback of this approach is that it requires an initial “discovery” run to identify abundant components to be excluded subsequently and thus necessitates additional material (which may pose a problem when working with small human biopsies) and comes at a higher cost, which may limit the applicability of such approach.

## Translational Implications of ECM Proteomics Studies: a FOCUS ON Cancer

Having overcome inherent challenges in studying the ECM and having established the feasibility of analyzing the protein composition of the ECM using bottom-up proteomics, the question became: can we derive meaningful biological and clinical information from ECM proteomic studies?

Since the ECM plays key roles in maintaining the homeostasis of all physiological systems, dysregulation of the ECM has consequences on all those systems. Over the past decade, ECM proteomics has thus been applied to a vast range of samples (*e.g.*, clinical biopsies, tissues from mouse models of human diseases, tissue interstitial fluid or serum, ECM produced by cells in culture) and in the context of diverse diseases and disorders. Excellent reviews have recently discussed the application of proteomics to study the ECM of the skin (98), cardiovascular diseases (99, 100), liver diseases (101, 102), lung diseases (103), and neurodegenerative diseases (64, 104, 105).

This section will thus focus on selected studies of the past 5 years that have employed matrisomics to study the ECM of cancer microenvironments. For a survey of early proteomic studies of the cancer matrisome (including melanoma, multiple myeloma, colorectal cancer), readers are invited to refer to our previous review (106).

### The Matrisome of Breast Cancers

In an early study, we applied ECM proteomics to characterize the protein composition of the ECM of poorly and highly metastatic primary mammary tumor xenografts. This study led to the identification of a 43-matrisome-protein signature characteristic of mammary tumors of higher metastatic potential (107). This signature included matrisome proteins that had previously been associated with breast cancer progression such as lysyl oxidase-like 2 (108) and angiopoietin-like 4 (109), and also identified known matrisome proteins that had never been associated with this disease before such as the latent TGFβ-binding protein 3 (LTBP3). It also identified a novel ECM glycoprotein of unknown function, SNED1. Importantly, we further demonstrated that the level of expression of *LTBP3* and *SNED1* negatively correlated with breast cancer patient prognosis (110). In a follow-up study, we employed TMT-based proteomics to compare the protein composition of the ECM of metastases arising from human mammary tumor cells in different organs, namely, the brain, lungs, liver, and bone marrow. This approach allowed us to delineate the contribution of tumor cells and stromal cells to the building of the metastatic niche, since human proteins secreted by tumor cells can be distinguished from murine proteins secreted by host cells (111). This study led to the quantification of over 300 human and mouse matrisome proteoforms produced by either or both tumor cells and stromal cells. We further showed that each metastatic niche was characterized by a unique ECM composition contributed by both tumor cells and stromal cells (111).

Work pioneered by the late Dr Patricia Keely revealed that remodeling of the architecture of the fibrillar collagen meshwork is a key driver of breast cancer progression (112, 113, 114). Over the past 5 years there has thus been an increased interest in applying ECM proteomics to characterize and identify the mechanisms driving these changes. Tomko and colleagues applied ECM proteomics to profile compositional changes accompanying the remodeling of the fibrillar collagen meshwork observed in invasive ductal carcinomas (115). They identified 27 matrisome proteins with differential abundance between healthy mammary tissue and invasive ductal carcinoma biopsies, including tenascin-C and thrombospondin-2 that presented a distribution pattern correlating with that of the remodeled fibrillar collagen meshwork in tumor samples. In a 2020 study, the Weaver laboratory employed ECM proteomics to identify protein signatures correlating with the four classes of mammographic densities according to the Breast Imaging–Reporting and Data System (116). The team found that samples from tissues with higher mammographic density were enriched for fibrillar collagens (type I and V) and the fibril-associated collagen type XII. Importantly, a higher mammographic density has been correlated with an increased life-time risk of breast malignancy.

The Cox laboratory recently published a study surveying changes in matrisome protein composition at different stages of tumor progression using a genetically engineered mouse model of breast cancer (117). The team reported the identification of four classes of proteins: the first one encompassing proteins detected with increased abundance over time in healthy mammary gland and with even higher abundance at different stages of tumor progression; the second consisting of proteins detected in higher abundance in tumors as compared with healthy tissues; the third including proteins with abundance negatively correlating with tumor progression; and the fourth representing proteins detected in decreased abundance in early and mid-stages of development as compared with healthy tissue but with increased abundance in late-stage tumors as compared with normal age-matched mammary glands (117). The team reported that collagen type XII strongly correlated with tumor progression and confirmed results of a prior study conducted in patients with breast cancer demonstrating an increased expression of *COL12A1* in breast cancers as compared with normal mammary tissue (116). The team further showed, using tissue microarray (TMA) staining on a cohort of 150 patients, that the abundance of collagen type XII negatively correlated with disease-specific and recurrence-free survival. This result demonstrates the robustness of data obtained using ECM proteomics on a small number of samples (n = 5) and the translational potential of findings obtained on preclinical models to patients. Mechanistically, the authors showed that collagen type XII is expressed by cancer-associated fibroblasts and contributes to create a permissive microenvironment supportive of cancer invasion by remodeling the architecture of the fibrillar collagen meshwork. This study opens up the possibility of using COL12A1 not only as a prognostic marker but also as a target for antimetastatic strategies.

Recent large-cohort studies have established that obesity is associated with an increased risk of cancer, including breast cancer (118). The Fischbach laboratory established that obesity could induce mammary ECM remodeling and, in turn, promote breast tumorigenesis in a mouse model (119). Based on this observation, the Oudin laboratory employed TMT proteomics to compare the ECM composition of mammary gland from mice fed a chow diet or a high-fat diet. The team reported the identification of a set of ECM proteins present in differential abundance between the two conditions, with collagen type XII presenting the highest abundance increase in the mammary gland of obese mice as compared with control (120). Comparison of the ECM proteins found enriched in obese mammary gland as compared with control mammary gland, in murine mammary tumors as compared with healthy mammary gland (121), and in highly metastatic *versus* poorly metastatic mammary tumor xenografts (107) and identified a 9-ECM-protein signature comprising collagens type VI and type XII, fibronectin, elastin, vitronectin, the laminin alpha 5 chain, annexin A3, galectin 1, and the von Willebrand Factor A Domain Containing 1 protein (120). This suggests that obesity-induced compositional changes in the mammary gland ECM overlaps with changes observed during cancer progression. The functional relevance and prognostic value of this signature was further exemplified by showing that collagen type VI promoted breast cancer invasiveness and that the expression level of the three genes COL6A1, COL6A2, and COL6A3, encoding the three chains assembling for the functional collagen type VI protein, negatively correlated with breast cancer patient prognosis (120).

Another study from the Oudin laboratory characterized the changes in the ECM composition of the mammary gland of mice that received chemotherapy (paclitaxel or doxorubicin) or a vehicle control (122). The authors showed that treatment with paclitaxel resulted in changes in ECM composition (5 matrisome proteins detected in higher abundance and 54 in lower abundance, as compared with vehicle control) distinct from the changes induced by doxorubicin treatment (32 matrisome proteins detected in higher abundance and 28 in lower abundance, as compared with vehicle control, with a marked decrease in the abundance of several collagens). The comparison of the two signatures identified two secreted factors S100a9 and trichohyalin in higher abundance upon treatment, while 11 matrisome proteins were detected in lower abundance upon treatment, including the core matrisome glycoproteins, periostin, thrombospondin 2, and hemicentin.

### The Matrisome of Pancreatic Ductal Adenocarcinomas

Pancreatic cancers accounted for close to 60,000 new cancer cases diagnosed in the United States in 2022 (123). With one of the worst 5-year survival rates (11%), pancreatic ductal adenocarcinoma (PDAC) is also one of the deadliest cancer types (124). This is because it tends to be diagnosed at late stages and only limited therapeutic interventions are available (125). PDAC is also one of the most fibrotic cancer types and is characterized by an excessive ECM accumulation, which, we now know, is a key factor driving cancer aggressiveness and resistance to treatment (126). Proteomic profiling of the ECM of genetically engineered mouse models of pancreatic ductal adenocarcinomas at different stages of tumor progression revealed that the early stage of PDAC progression was characterized by an increase in the abundance of the glycoprotein fibronectin, the matricellular proteins tenascin-C and thrombospondins 1 and 2, and the ECM cross-linking enzymes lysyl oxidase-like 2 and transglutaminase 2 and a concomitant decrease in the abundance of basement membrane components: alpha chains 1 and 2 of collagen IV and the chains composing the laminin trimer 221 (127). Further disease progression saw a decrease in matricellular proteins but an increase in several proteoglycans (fibromodulin, biglycan, prolargin) (127).

More recently, Tian and collaborators profiled the ECM composition of early-stage human pancreatic intraepithelial neoplasia and advanced PDAC samples using TMT proteomics and identified a subset of 136 matrisome proteins, including collagen type I, fibronectin, fibrillin 1, and periostin, present in high abundance in pancreatic intraepithelial neoplasias as compared with healthy pancreatic tissue, and in even higher abundance in PDAC samples (128). Moreover, using xenograft models, Tian and collaborators demonstrated that over 90% of the matrisome proteins of the PDAC microenvironment were secreted by stromal cells, while 10% were secreted by tumor cells. In a follow-up study, the team demonstrated that three of the proteins secreted by the tumor cells, agrin, SERPINB5, and cystatin B, promoted PDAC metastasis and correlated with poor patient prognosis (129).

### ECM Signatures of Distinct Steps of Cancer Progression

Most cancer types undergo a stepwise progression. One of the key steps in cancer progression is angiogenesis, the step at which tumors become vascularized (130). This step is critical for tumor growth through the increased availability of oxygen and nutrients and for tumor dissemination *via* the systemic circulation (131). Using label (iTRAQ)-based quantitative proteomics and a genetically engineered mouse model recapitulating insulinoma progression and characterized by a precisely defined angiogenic switch (132), we profiled the ECM composition of nonangiogenic and angiogenic tumors. We found that the core matrisome proteins periostin and the EGF-containing fibulin extracellular matrix protein 1 (Efemp1) increased in abundance, while decorin, hemicentin, DMBT1, and the von Willebrand factor A domain containing protein 5A (VWA5A) decreased in abundance during the angiogenic switch (133).

Dormancy is another critical step of cancer progression. During this phase, tumor cells that have disseminated to distant organs enter quiescence prior to reawakening, which leads to patient relapse, sometimes decades later (134). Recent studies have pointed to the role of the local and systemic environments in the maintenance of dormancy or, on the contrary, in the reactivation of a proliferative program (135, 136). Yet, the precise mechanisms by which the ECM influences this key step in cancer progression are not known. In a 2022 study, we reported the characterization of the ECM of a mouse model of proliferative (T-Hep3) and dormant (D-Hep3) head and neck squamous cell carcinoma xenografts and identified collagen type III as produced in higher abundance by dormant tumor cells (137). We further showed that re-expression of *COL3A1*, encoding collagen type III, restored the proliferative potential of tumor cells *via* the discoidin domain receptor 1 and the Stat signaling pathway. Mechanistically, we showed that the reactivation or “awakening” of tumor cells upon collagen type III production was supported by a change in the architecture of the fibrillar collagen meshwork within the ECM (137). This study strongly supports the idea that manipulating the tumor microenvironment may be a viable approach to preventing metastatic growth and relapse.

### ECM Signatures of Cancer Subtypes

Tumors arising in a given organ can originate from different cell types and/or in different regions in that organ and hence may contain a different ECM. Proteomics has been applied to study the ECM composition of two of the main common types of brain tumors, medulloblastomas and glioblastomas (GBMs), and has revealed that, in addition to unique ECM signatures distinguishing medulloblastomas and glioblastomas, a subset of ECM proteins was detected in higher abundance in both tumor types as compared with control brain tissues (138). In a recent study, Sethi *et al.* applied glycoproteomics and glycomics to investigate the ECM composition (glycoproteins and GAG moieties of proteoglycans, respectively) of different human glioblastoma subtypes with the goal of identifying potential GBM biomarkers (139). This study employed an interesting protocol, using a TMA of fixed GBM and control samples as starting material. Presumably, the limited size of the starting material did not allow sample decellularization, but using a chemical inkjet printer, the team was able to “print” on each TMA core a combination of enzymes (*e.g.*, deglycosylating enzymes, proteinases) and derive the matrisome profile of each sample. The study identified a total of 146 matrisome components and revealed differences in the nature of the GAGs detected, in the nature and abundance of ECM proteins detected, and also, interestingly, in the nature and abundance of certain types of posttranslational modifications (*e.g.*, proline hydroxylation of collagen peptides) between the different GBM subtypes. Since the study was conducted on intact tissue samples, the team was also able to detect changes in the abundance of intracellular enzymes responsible for the PTMs of ECM proteins (*e.g.*, glycosyltransferase and glycosidase enzymes), something that cannot be captured when working on decellularized samples.

In summary, ECM-focused proteomics is now broadly adopted to study the composition of tumor microenvironments and to identify proteins characteristic of disease stages. As recent studies have shown, matrisome proteins whose change in abundance correlates with disease progression can serve as prognostic markers or be used to monitor treatment efficiency. Interestingly, certain ECM proteins or families of proteins (matricellular proteins such as tenascin-C, thrombospondins, periostin; fibrillar and fibril-associated collagens like collagens type I, III, and XII; ECM cross-linking enzymes of the lysyl oxidase and transglutaminase families) are consistently altered across different cancer types, opening the possibility of the existence of common ECM-dependent mechanisms being at play during tumor progression. This is of paramount importance as we think about ways to exploit the ECM to develop new anticancer strategies.

## The Next Frontiers in ECM Proteomics

### Capturing ECM Proteoform Diversity

Once challenging, the characterization of the protein composition of the ECM of tissues is now broadly accessible. But ECM proteins, beyond their identity, have much more to reveal. For example, to what extent are they posttranslationally modified? Do ECM PTMs vary during the time course of disease progression or with aging? How are ECM proteins folded? With which other proteins do they interact? Addressing these questions will contribute to increasing our knowledge of ECM protein functions and of the fundamental mechanisms orchestrated by cell–ECM interactions. MS-based proteomics has the potential to help address these questions.

The recognition of proteoform diversity is not recent (140), neither is the recognition of the need to profile proteoforms (141, 142). Examples of key roles played by ECM proteoforms abound in the context of cancer: proteoforms arising from alternative splicing of fibronectin or tenascin-C are specifically detected in tumors but not healthy tissues and have been shown to promote tumor progression (143, 144, 145). Disease-specific ECM proteoforms have thus been proposed to be excellent candidates to mediate the targeted delivery of imaging or therapeutic agents to diseased tissues (31,35, 146). Yet, current ECM proteomics approaches result, with the exception of the most abundant ECM proteins, like collagen type I, in modest sequence coverage, limiting our ability to capture proteoform diversity. For example, interrogation of the ECM protein knowledge database, MatrisomeDB (147), reveals that the coverage of fibronectin (UniProt P11276) is 20.6% in healthy liver samples or 11.5% in primary metastatic colorectal tumor samples, and that of thrombospondin1 (UniProt P07996) 10.9% in the ECM of normal saphenous vein samples.

In addition to proteoforms resulting from alternative splicing, ECM proteins can include single amino acid variants. For example, we have recently shown that, in most cancers, matrisome genes are mutated with higher frequency than nonmatrisome genes (148). Importantly, we have further shown that, in certain cases, mutations in ECM genes correlated with patient survival (148). The Polyak laboratory has also recently demonstrated that there is an enrichment in somatic mutations in matrisome genes during the transition of ductal carcinoma *in situ* to invasive ductal carcinoma (149). However, the functional impact of these mutations remains unknown, and we are yet to identify products of mutated genes in cancer samples.

The main process leading to proteoform diversity is *via* posttranslational modification. For ECM proteins, this process can occur both intracellularly and extracellularly and has been shown to contribute to different pathophysiological processes. For example, increased collagen cross-linking resulting in a stiffer ECM is a hallmark of cancer (150); fibronectin citrullination increases cell migration *in vitro* and *in vivo* in the context of wound healing (151); lysine acetylation of fibronectin is involved in renal fibrosis (152). ECM proteins can also be phosphorylated. However, the kinases responsible for the phosphorylation of secreted proteins have only recently been discovered (153, 154, 155, 156) and most ECM proteomic studies, including our own, did not allow phosphorylations of serine, threonine, and tyrosine as variable modifications. Current strategies aimed at enriched posttranslationally modified proteins (157) cannot be readily applied to study the ECM because of the insoluble nature of ECM proteins. It is thus becoming obvious that strategies to increase protein sequence coverage (*e.g.*, protein and peptide fractionation), as well as the enrichment of protein databases used to search LC-MS/MS output with patient-specific sequences derived from the large body of RNA-Seq studies, and broader or even open search strategies, should contribute to enhance our ability to detect and quantify ECM proteoforms. As a community, we will also need novel analytical methods to interpret LC-MS/MS output. For example, Merl-Pham and colleagues have reported the development of a pipeline aimed at mapping and quantifying proline and lysine hydroxylations and lysine glycosylations for 15 collagen chains produced *in vitro* by primary lung fibroblasts isolated from patients with idiopathic lung fibrosis (158).

Proteolytic cleavage is an important mechanism leading to matrisome proteoforms diversity. ECM protein degradation by matrix metalloproteinases (159, 160), a disintegrin and metalloproteases (ADAM) (161), a disintegrin-like and metalloproteinase domain with thrombospondin-type 1 motifs proteins (ADAMTS) (162), or cathepsin (163) plays key roles in pathophysiological processes (15). In addition, cleavage fragments of ECM proteins, known as matrikines (or matricryptins) (164), exert signaling functions, sometimes distinct from that of the proteins they arise from (165, 166). For example, endostatin is a naturally occurring cleavage fragment of type XVIII collagen, arresten and tumstatin are cleavage fragments of type IV collagen, and all have antiangiogenic properties (167). Identifying proteolytic fragments of proteins using standard proteomics is challenging. To overcome this, novel MS-based methods grouped under the term of “degradomics” targeting the investigation of protein N termini and protease substrates have been devised (168). In recent years, ECM degradomics has become an active field of investigation (68, 169, 170), in particular in musculoskeletal and dermatology research (171, 172, 173, 174, 175).

Studying the protein components of the ECM only gives a partial view of this complex compartment. Indeed, most ECM proteins are glycosylated, and, in the case of proteoglycans (*e.g.*, perlecan, decorin, versican) (176), the protein moiety only constitutes the minor portion of these components, with GAGs such as chondroitin sulfate, dermatan sulfate, and keratan sulfate composing their majority. MS-based glycomics and glycoproteomics are powerful approaches to characterize the glycans conjugated to proteins (67, 177, 178, 179). These approaches have begun to be applied to the study of the ECM (64) and have led to the identification of glycosylation sites and the characterization of the nature of GAG (180).

Lastly, posttranslational modifications that accumulate over time can alter protein folding and this can impact the nature and quantity of peptides detected by LC-MS/MS. We can thus envision being able to leverage ECM proteomics to gain structural information on ECM proteins. To this end, Eckersley and colleagues have developed a peptide mapping strategy called “peptide location fingerprinting” to identify structural changes, approximated by peptide yields, in ECM proteins (181, 182, 183). By reinterrogating previously published datasets on the ECM of aging mouse lung, aging human intervertebral discs, and atherosclerotic arteries, the team identified proteins displaying conserved structural differences in healthy *versus* altered states (181, 184).

### Toward a More Accurate Quantification of Matrisome Protein Abundance

The field of ECM proteomics has not yet broadly adopted targeted assays. This is not unexpected as target identification is a prerequisite to designing such assays. Yet, being able to quantify the abundance of ECM proteins across different conditions more accurately is a first step toward understanding the mechanisms leading to ECM dis-homeostasis in disease, and a necessary step toward the development of interventional strategies aimed at modulating the ECM to revert or correct disease-associated phenotypes. With significant advances made over the past decade, our field is now mature and ready to enter the next phase and employ targeted and more quantitative approaches.

One such approach uses QConCATs, which are standard peptides concatenated in artificial polypeptides, allowing the precise quantification of a limited number of target proteins (185, 186). The Hansen lab has developed QConCATs to quantify 77 matrisome proteins and successfully applied this strategy to profile the ECM of the mammary gland and liver microenvironments (85).

Selective or multiple reaction monitoring (187) is another approach permitting the quantification, with high accuracy, of a subset of proteins of interest. As of today, such approaches have not been applied to ECM research. Yet, resources are available. Of the 3500 validated assays available in October 2022 in the National Cancer Institute’s Clinical Proteomic Tumor Analysis Consortium (CPTAC) portal (188, 189), 359, or approximately 10%, are designed to quantify 169 human or murine matrisome proteins: 48 ECM glycoproteins, 8 collagens, 7 proteoglycans for the core matrisome and 29 ECM-affiliated proteins, 71 ECM regulators, 28 secreted factors, for matrisome-associated proteins (supplemental Table S1). Global ECM proteomics, by permitting the identification of proteins of interest, has thus laid the foundations of future targeted studies.

### Building Spatially Resolved Maps of the ECM

The first step of all sample preparation protocols described above consists of the mechanical disruption of tissues. Any information regarding the spatial distribution of ECM proteins within tissues and organs is thus lost (190). Yet, we know that ECM proteins are not homogeneously distributed across tissues. For example, the specialized basement membrane ECM surrounding blood vessels or underlying epithelia is different in composition, structure, and functions than the interstitial ECM found in connective tissues. Knowledge of the patterns of distribution and organization of ECM proteins *in situ* can thus provide crucial information on their functions. Certain structures within organs can be physically isolated to allow for a more specific ECM analysis. For example, treatment of murine pancreata by collagenase releases pancreatic islets that can further be processed for matrisome profiling (133). The Lennon laboratory has employed laser capture microdissection to isolate glomeruli from kidneys for analysis of the glomerular basement membrane ECM (191). A similar approach was adopted by Paunas and colleagues to identify ECM changes in the glomerular basement membrane of patients diagnosed with IgA nephropathy (192). Using laser capture microdissection of decellularized skin samples to isolate six distinct regions of the skin, Li and colleagues have defined the first spatially resolved map of the skin matrisome and have shown that each region is characterized by a unique ECM signature and by distinct cellular programs (94). Using the spatial separation of different brain regions and precise dissection, the Götz laboratory compared the ECM composition of the nonneurogenic somatosensory cortex, the olfactory bulb, the lateral subependymal zone where most neural stem cells reside, and the medial subependymal zone and identified a subset of matrisome proteins enriched in the neural stem cell niche of adult murine brain samples (83).

While these approaches allow the analysis of defined structures or regions within organs and tissues, we are still lacking the tools to build spatially resolved maps of the ECM at the cellular resolution. Progress may come from another MS-based technology, imaging mass spectrometry (IMS). IMS is commonly applied to the study of lipids, glycans, and metabolites (193) but has recently been applied for the first time to define the distribution pattern of ECM proteins within tissues (69, 70, 193). IMS, however, presents limitations: it has a narrow dynamic range and ECM proteins may not be good candidates for IMS due to their highly cross-linked nature, which may hinder ionization. Leveraging LC-MS/MS data, multiplexed antibody-based approaches can also be envisioned (*e.g.*, CODEX (190)). Significant efforts are being put toward the validation of antibodies, for example, through the Human Protein Atlas (194) or the production of Organ Mapping Antibody Panels by the Human BioMolecular Atlas Program (195). ECM proteins should not be forgotten in these efforts, since they provide the context in which cells function.

## Resources for ECM Proteomics Research

The emergence of MS-based proteomics as the method of choice to study the ECM has been concurrent to the emergence of principles dictating “Findability, Accessibility, Interoperability, and Reuse” (FAIR) of -omic datasets (196). This has permitted the development and expansion of ECM knowledgebases and databases briefly described here and summarized in Table 1.

**Table 1:** Resources for ECM proteomic research

Resource	Description	URL	Reference
The Matrisome Project website	Web portal hosting resources for ECM -omic research	https://matrisome.org	
MatrisomeDB	Searchable database compiling ECM proteomic datasets reprocessed using a unified search, quality-control, and analysis pipeline. Species included: *Homosapiens*, *Mus musculus*	https://matrisomedb.org	Shao et al., 2023 (147)
Manchester Proteome Location Fingerprinting	Allows users to find locational differences in peptide yields from ECM proteins across experimental conditions and tissues (skin, blood vessels, tendons, intervertebral discs) Species included: *H. sapiens*	https://www.manchesterproteome.manchester.ac.uk/#/MPLF	Ozols *et al.*, 2021 (183)
Termini-oriented protein function inferred database (TopFIND)	Knowledgebase of proteases, protease substrates, and N- and C- terminomic datasets Species of relevance to ECM research included: *H. sapiens*, *M. musculus*, *Rattus norvegicus*, *Caenorhabditis elegans*, *Danio rerio*	https://topfind.clip.msl.ubc.ca/	Fortelny *et al.*, 2015 (200)
MatrixDB	Database reporting curated interactions established by ECM proteins, proteoglycans, and polysaccharides Species included: *H. sapiens*	http://matrixdb.univ-lyon1.fr/	Clerc *et al.*, 2019 (202)
MatriNet	Database enabling the investigation of tumor-specific and pan-cancer ECM gene network modules and differential gene interaction patterns Species included: *H. sapiens*	https://www.matrinet.org/	Kontio *et al.*, 2022 (203)
basement membraneBASE	Knowledgebase of basement membrane composition during development, adult life, disease, and across animal species. Provides list of basement membrane genes and protein localization data based on antibody evidence or endogenously tagged genes Species included: *H. sapiens*, *M. musculus*, *R. norvegicus*, *Bos taurus*, *Oryctolagus cuniculus*, *Canis familiaris*, *Gallus gallus*, *D. rerio*, *Drosophila melanogaster*, *C. elegans*	https://bmbasedb.manchester.ac.uk/	Jayadev *et al.*, 2022 (204)

### MatrisomeDB and the ECM Atlas

Witnessing the adoption of proteomics to study the ECM, we released in 2016 a draft of an ECM atlas, including ECM proteomics datasets from 14 different tissue and tumor types and made the data available through a searchable database (197). We expanded our efforts and released in 2019 MatrisomeDB (https://matrisomedb.org/), the ECM protein knowledge database, which included curated ECM datasets from 17 published studies reprocessed using unified search parameters and available through an interface allowing multiple query inputs (*e.g.*, gene symbol, protein name, protein signature, matrisome category, tissue type, species) (198). The latest release of MatrisomeDB includes datasets from 42 curated ECM proteomic studies, providing data on 2051 human and 949 mouse matrisome proteoforms identified from 6,891,623 human and 4,763,174 mouse matrisome-protein-derived peptide-to-spectrum matches (147). Novel functionalities implemented in this release include enhanced peptide and PTM mapping on domain-based representations and 3D structures of matrisome proteins, as well as referencing to external databases such as the CPTAC assay portal (188) and the Peptide Atlas (199) to facilitate the application of targeted MS to the study of matrisome proteins (147).

### The Manchester Peptide Location Fingerprinting

The Manchester Peptide Location Fingerprinting developed by the laboratory of Dr Michael Sherratt at the University of Manchester (https://www.manchesterproteome.manchester.ac.uk/#/MPLF) allows the user to query preanalyzed datasets with the goal of identifying structural alterations of ECM proteins, resulting in differential peptide yields in proteomic datasets, across pathophysiological conditions (183).

### TopFIND

Developed by the laboratory of Dr Overall, the Termini-oriented protein Function Inferred Database (TopFIND) is a database of protease cleavage sites, protein termini, and protein terminus modifications (https://topfind.clip.msl.ubc.ca/) (200). While this resource is not exclusively dedicated to ECM research, users can interrogate TopFIND for cleavage fragment and neotermini identified or inferred for their favorite ECM proteins *via* the “protein” interface.

### MatrixDB: the ECM Interaction Database

A key to understanding ECM roles in pathophysiological processes is to decipher the nature of ECM protein interactions with each other, which lead to the assembly of the ECM scaffold, and also with cells. However, the insoluble nature of matrisome proteins significantly limits our availability to employ strategies used in other contexts to study protein interactomes, such as immune precipitation coupled to MS (201). The Ricard-Blum laboratory developed MatrixDB (http://matrixdb.univ-lyon1.fr/), a database reporting ECM protein–ECM protein and ECM protein–glycan interactions validated experimentally *in vitro* and allowing the building of interaction networks. However, to interact, proteins need to be present in the same tissue with precise stoichiometry. To account for this, we integrated the content of MatrisomeDB to the 2019 release of the MatrixDB, allowing users to build tissue-specific interaction networks (202).

### MatriNet

MatriNet (https://www.matrinet.org/) was developed by the Izzi laboratory at the University of Oulu to study the ECM connectome and ECM networks in normal and cancerous tissues (203). Originally developed to mine transcriptomic datasets and identify coregulated ECM genes and pathways, MatriNet can also leverage proteomic profiles to identify features of ECM networks across pathophysiological conditions.

### Basement Membranebase

Basement membraneBASE (https://bmbase.manchester.ac.uk/) is a knowledgebase jointly developed by the University of Manchester and Duke University that provides information on the composition of the specialized basement membrane ECM in development, life, and disease and across multiple species (204). It also includes information on matrisome protein localization from immunohistochemical staining experiments and provides users with resources, including a list of antibodies and protocols, to study basement membrane proteins.

## Conclusion

A better understanding of the biochemical properties of ECM proteins has led, over the past decade, to the development of MS-based strategies capable of decoding the compositional complexity of the ECM of tissues. The democratization of such strategies has resulted in the definition of the matrisome of many tissues, across diverse pathophysiological states, and in the identification of ECM signatures characteristics of these states, leading, in some cases, to the discovery of novel biomarkers of human diseases such as cancer. The ECM holds the promise to be an important reservoir of novel biomarkers and therapeutic targets (205, 206). In 2016, we had postulated that the ECM was ready to enter the -omic era (207). With the significant advances made over the past years, and successful examples from preclinical studies, ECM proteomic research is ready for a new chapter and can now enter the clinical proteomic era.

## Supplemental data

This article contains supplemental data.

## Conflict of interest

A. N. has sponsored research agreements with Boehringer-Ingelheim.

## References

[1] Hynes R.O. (2012). The evolution of metazoan extracellular matrix. J. Cell Biol..

[2] Adams J.C., Keeley F.W., Mecham R.P. (2013). Evolution of Extracellular Matrix.

[3] Karamanos N.K., Theocharis A.D., Piperigkou Z., Manou D., Passi A., Skandalis S.S. (2021). A guide to the composition and functions of the extracellular matrix. FEBS J..

[4] Humphrey J.D., Dufresne E.R., Schwartz M.A. (2014). Mechanotransduction and extracellular matrix homeostasis. Nat. Rev. Mol. Cell Biol..

[5] Dooling L.J., Saini K., Anlaş A.A., Discher D.E. (2022). Tissue mechanics coevolves with fibrillar matrisomes in healthy and fibrotic tissues. Matrix Biol..

[6] Kanchanawong P., Calderwood D.A. (2022). Organization, dynamics and mechanoregulation of integrin-mediated cell–ECM adhesions. Nat. Rev. Mol. Cell Biol..

[7] Liebscher I., Cevheroğlu O., Hsiao C.C., Maia A.F., Schihada H., Scholz N. (2021). A guide to adhesion GPCR research. FEBS J..

[8] Yamada K.M., Collins J.W., Cruz Walma D.A., Doyle A.D., Morales S.G., Lu J. (2019). Extracellular matrix dynamics in cell migration, invasion and tissue morphogenesis. Int. J. Exp. Pathol..

[9] Dzamba B.J., DeSimone D.W. (2018). Extracellular matrix (ECM) and the sculpting of embryonic tissues. Curr. Top Dev. Biol..

[10] Karamanos N.K., Theocharis A.D., Neill T., Iozzo R.V. (2019). Matrix modeling and remodeling: a biological interplay regulating tissue homeostasis and diseases. Matrix Biol..

[11] Lausecker F., Lennon R., Randles M.J. (2022). The kidney matrisome in health, aging, and disease. Kidney Int..

[12] Ewald C.Y. (2020). The matrisome during aging and longevity: a systems-level approach toward defining matreotypes promoting healthy aging. Gerontology.

[13] Lamandé S.R., Bateman J.F. (2020). Genetic disorders of the extracellular matrix. Anat. Rec. (Hoboken)..

[14] Lu P., Takai K., Weaver V.M., Werb Z. (2011). Extracellular matrix degradation and remodeling in development and disease. Cold Spring Harb. Perspect. Biol..

[15] Bonnans C., Chou J., Werb Z. (2014). Remodelling the extracellular matrix in development and disease. Nat. Rev. Mol. Cell Biol..

[16] Theocharis A.D., Manou D., Karamanos N.K. (2019). The extracellular matrix as a multitasking player in disease. FEBS J..

[17] Malfait F., Castori M., Francomano C.A., Giunta C., Kosho T., Byers P.H. (2020). The Ehlers-Danlos syndromes. Nat. Rev. Dis. Primers.

[18] Schulz J.N., Plomann M., Sengle G., Gullberg D., Krieg T., Eckes B. (2018). New developments on skin fibrosis - essential signals emanating from the extracellular matrix for the control of myofibroblasts. Matrix Biol..

[19] Bruckner-Tuderman L., Has C. (2014). Disorders of the cutaneous basement membrane zone--the paradigm of epidermolysis bullosa. Matrix Biol..

[20] Cook J.R., Carta L., Galatioto J., Ramirez F. (2015). Cardiovascular manifestations in Marfan syndrome and related diseases; multiple genes causing similar phenotypes. Clin. Genet..

[21] Zhou Y., Horowitz J.C., Naba A., Ambalavanan N., Atabai K., Balestrini J. (2018). Extracellular matrix in lung development, homeostasis and disease. Matrix Biol..

[22] Bülow R.D., Boor P. (2019). Extracellular matrix in kidney fibrosis: more than just a scaffold. J. Histochem. Cytochem..

[23] Chew C., Lennon R. (2018). Basement membrane defects in genetic kidney diseases. Front. Pediatr..

[24] Pakshir P., Hinz B. (2018). The big five in fibrosis: macrophages, myofibroblasts, matrix, mechanics, and miscommunication. Matrix Biol..

[25] Pickup M.W., Mouw J.K., Weaver V.M. (2014). The extracellular matrix modulates the hallmarks of cancer. EMBO Rep..

[26] Cox T.R. (2021). The matrix in cancer. Nat. Rev. Cancer.

[27] Winkler J., Abisoye-Ogunniyan A., Metcalf K.J., Werb Z. (2020). Concepts of extracellular matrix remodelling in tumour progression and metastasis. Nat. Commun..

[28] Souza da Silva R.M., Queiroga E.M., Paz A.R., Neves F.F.P., Cunha K.S., Dias E.P. (2021). Standardized assessment of the tumor-stroma ratio in colorectal cancer: interobserver validation and reproducibility of a potential prognostic factor. Clin. Pathol..

[29] van Pelt G.W., Sandberg T.P., Morreau H., Gelderblom H., van Krieken J.H.J.M., Tollenaar R.A.E.M. (2018). The tumour-stroma ratio in colon cancer: the biological role and its prognostic impact. Histopathology.

[30] Cardoso F., van’t Veer L.J., Bogaerts J., Slaets L., Viale G., Delaloge S. (2016). 70-Gene signature as an aid to treatment decisions in early-stage breast cancer. N. Engl. J. Med..

[31] Jailkhani N., Ingram J.R., Rashidian M., Rickelt S., Tian C., Mak H. (2019). Noninvasive imaging of tumor progression, metastasis, and fibrosis using a nanobody targeting the extracellular matrix. Proc. Nat. Acad. Sci. U. S. A..

[32] Santimaria M., Moscatelli G., Viale G.L., Giovannoni L., Neri G., Viti F. (2003). Immunoscintigraphic detection of the ED-B domain of fibronectin, a marker of angiogenesis, in patients with cancer. Clin. Cancer Res..

[33] Steiner M., Neri D. (2011). Antibody-radionuclide conjugates for cancer therapy: historical considerations and new trends. Clin. Cancer Res..

[34] Pasche N., Neri D. (2012). Immunocytokines: a novel class of potent armed antibodies. Drug Discov. Today.

[35] Lieverse R.I.Y., Van Limbergen E.J., Oberije C.J.G., Troost E.G.C., Hadrup S.R., Dingemans A.M.C. (2020). Stereotactic ablative body radiotherapy (SABR) combined with immunotherapy (L19-IL2) *versus* standard of care in stage IV NSCLC patients, ImmunoSABR: a multicentre, randomised controlled open-label phase II trial. BMC Cancer.

[36] Momin N., Mehta N.K., Bennett N.R., Ma L., Palmeri J.R., Chinn M.M. (2019). Anchoring of intratumorally administered cytokines to collagen safely potentiates systemic cancer immunotherapy. Sci. Transl. Med..

[37] Nyström A., Bernasconi R., Bornert O. (2018). Therapies for genetic extracellular matrix diseases of the skin. Matrix Biol..

[38] Schuppan D., Ashfaq-Khan M., Yang A.T., Kim Y.O. (2018). Liver fibrosis: direct antifibrotic agents and targeted therapies. Matrix Biol..

[39] Bejarano L., Jordāo M.J.C., Joyce J.A. (2021). Therapeutic targeting of the tumor microenvironment. Cancer Discov..

[40] Hauge A., Rofstad E.K. (2020). Antifibrotic therapy to normalize the tumor microenvironment. J. Transl. Med..

[41] Lampi M.C., Reinhart-King C.A. (2018). Targeting extracellular matrix stiffness to attenuate disease: from molecular mechanisms to clinical trials. Sci. Transl. Med..

[42] Ley K., Rivera-Nieves J., Sandborn W.J., Shattil S. (2016). Integrin-based therapeutics: biological basis, clinical use and new drugs. Nat. Rev. Drug Discov..

[43] Wilson R. (2010). The extracellular matrix: an underexplored but important proteome. Expert Rev. Proteomics.

[44] Filipe E.C., Chitty J.L., Cox T.R. (2018). Charting the unexplored extracellular matrix in cancer. Int. J. Exp. Pathol..

[45] Rappu P., Salo A.M., Myllyharju J., Heino J. (2019). Role of prolyl hydroxylation in the molecular interactions of collagens. Essays Biochem..

[46] Buehler M.J. (2006). Nature designs tough collagen: explaining the nanostructure of collagen fibrils. Proc. Natl. Acad. Sci. U. S. A..

[47] Shoulders M.D., Raines R.T. (2009). Collagen structure and stability. Annu. Rev. Biochem..

[48] Schwarzbauer J.E., DeSimone D.W. (2011 Jul 1). Fibronectins, their fibrillogenesis, and *in vivo* functions. Cold Spring Harb. Perspect. Biol..

[49] Ozsvar J., Yang C., Cain S.A., Baldock C., Tarakanova A., Weiss A.S. (2021). Tropoelastin and elastin assembly. Front. Bioeng. Biotechnol..

[50] Ricard-Blum S. (2011). The collagen family. Cold Spring Harb. Perspect. Biol..

[51] Wilson R., Bateman J.F. (2008). Cartilage proteomics: challenges, solutions and recent advances. Proteomics Clin. Appl..

[52] Lammi M.J., Häyrinen J., Mahonen A. (2006). Proteomic analysis of cartilage- and bone-associated samples. Electrophoresis.

[53] Hattar R., Maller O., McDaniel S., Hansen K.C., Hedman K.J., Lyons T.R. (2009). Tamoxifen induces pleiotrophic changes in mammary stroma resulting in extracellular matrix that suppresses transformed phenotypes. Breast Cancer Res..

[54] Wilson R., Diseberg A.F., Gordon L., Zivkovic S., Tatarczuch L., Mackie E.J. (2010). Comprehensive profiling of cartilage extracellular matrix formation and maturation using sequential extraction and label-free quantitative proteomics. Mol. Cell Proteomics.

[55] Belluoccio D., Wilson R., Thornton D.J., Wallis T.P., Gorman J.J., Bateman J.F. (2006). Proteomic analysis of mouse growth plate cartilage. Proteomics.

[56] Hansen K.C., Kiemele L., Maller O., O’Brien J., Shankar A., Fornetti J. (2009). An in-solution ultrasonication-assisted digestion method for improved extracellular matrix proteome coverage. Mol. Cell Proteomics.

[57] Didangelos A., Yin X., Mandal K., Baumert M., Jahangiri M., Mayr M. (2010). Proteomics characterization of extracellular space components in the human aorta. Mol. Cell Proteomics.

[58] Didangelos A., Yin X., Mandal K., Saje A., Smith A., Xu Q. (2011). Extracellular matrix composition and remodeling in human abdominal aortic aneurysms: a proteomics approach. Mol. Cell Proteomics.

[59] Chen W.C.W., Wang Z., Missinato M.A., Park D.W., Long D.W., Liu H.J. (2016). Decellularized zebrafish cardiac extracellular matrix induces mammalian heart regeneration. Sci. Adv..

[60] Garcia-Puig A., Mosquera J.L., Jiménez-Delgado S., García-Pastor C., Jorba I., Navajas D. (2019). Proteomics analysis of extracellular matrix remodeling during zebrafish heart regeneration. Mol. Cell Proteomics.

[61] Kessels M.Y., Huitema L.F.A., Boeren S., Kranenbarg S., Schulte-Merker S., Leeuwen JL van (2014). Proteomics analysis of the zebrafish skeletal extracellular matrix. PLoS One.

[62] Sessions A.O., Kaushik G., Parker S., Raedschelders K., Bodmer R., Van Eyk J.E. (2017). Extracellular matrix downregulation in the Drosophila heart preserves contractile function and improves lifespan. Matrix Biol..

[63] Sonpho E., Mann F.G., Levy M., Ross E.J., Guerrero-Hernández C., Florens L. (2021). Decellularization Enables characterization and functional analysis of extracellular matrix in planarian regeneration. Mol. Cell Proteomics.

[64] Raghunathan R., Sethi M.K., Klein J.A., Zaia J. (2019). Proteomics, glycomics, and glycoproteomics of matrisome molecules. Mol. Cell Proteomics.

[65] de Haan N., Pučić-Baković M., Novokmet M., Falck D., Lageveen-Kammeijer G., Razdorov G. (2022). Developments and perspectives in high-throughput protein glycomics: enabling the analysis of thousands of samples. Glycobiology.

[66] Kellman B.P., Lewis N.E. (2021). Big-data glycomics: tools to connect glycan biosynthesis to extracellular communication. Trends Biochem. Sci..

[67] Riley N.M., Bertozzi C.R., Pitteri S.J. (2021). A pragmatic guide to enrichment strategies for mass spectrometry-based glycoproteomics. Mol. Cell Proteomics.

[68] Haack A.M., Overall C.M., auf dem Keller U. (2022). Degradomics technologies in matrisome exploration. Matrix Biol..

[69] Angel P.M., Comte-Walters S., Ball L.E., Talbot K., Mehta A., Brockbank K.G.M. (2018). Mapping extracellular matrix proteins in formalin-fixed, paraffin-embedded tissues by MALDI imaging mass spectrometry. J. Proteome Res..

[70] Clift C.L., Drake R.R., Mehta A., Angel P.M. (2021). Multiplexed imaging mass spectrometry of the extracellular matrix using serial enzyme digests from formalin-fixed paraffin-embedded tissue sections. Anal. Bioanal. Chem..

[71] Naba A., Clauser K.R., Hoersch S., Liu H., Carr S.A., Hynes R.O. (2012). The matrisome: *in silico* definition and *in vivo* characterization by proteomics of normal and tumor extracellular matrices. Mol. Cell Proteomics.

[72] Hohenester E., Engel J. (2002 Mar 1). Domain structure and organisation in extracellular matrix proteins. Matrix Biol..

[73] Cromar G.L., Xiong X., Chautard E., Ricard-Blum S., Parkinson J. (2012). Toward a systems level view of the ECM and related proteins: a framework for the systematic definition and analysis of biological systems. Proteins: Struct. Funct. Bioinformat..

[74] Naba A., Hoersch S., Hynes R.O. (2012). Towards definition of an ECM parts list: an advance on GO categories. Matrix Biol..

[75] Hynes R.O., Naba A. (2012). Overview of the matrisome--an inventory of extracellular matrix constituents and functions. Cold Spring Harb. Perspect. Biol..

[76] Gebauer J.M., Naba A., Ricard-Blum S. (2020). Extracellular Matrix Omics [Internet].

[77] Martin G.R., Kleinman H.K., Terranova V.P., Ledbetter S., Hassell J.R. (1984). The regulation of basement membrane formation and cell-matrix interactions by defined supramolecular complexes. Ciba Found. Symp..

[78] Naba A., Clauser K.R., Hynes R.O. (2015). Enrichment of extracellular matrix proteins from tissues and digestion into peptides for mass spectrometry analysis. J. Vis. Exp..

[79] Naba A., Pearce O.M.T., Del Rosario A., Ma D., Ding H., Rajeeve V. (2017). Characterization of the extracellular matrix of normal and diseased tissues using proteomics. J. Proteome Res..

[80] Schiller H.B., Fernandez I.E., Burgstaller G., Schaab C., Scheltema R.A., Schwarzmayr T. (2015). Time- and compartment-resolved proteome profiling of the extracellular niche in lung injury and repair. Mol. Syst. Biol..

[81] Schiller H.B., Mayr C.H., Leuschner G., Strunz M., Staab-Weijnitz C., Preisendörfer S. (2017). Deep proteome profiling reveals common prevalence of MZB1-positive plasma B cells in human lung and skin fibrosis. Am. J. Respir. Crit. Care Med..

[82] Angelidis I., Simon L.M., Fernandez I.E., Strunz M., Mayr C.H., Greiffo F.R. (2019). An atlas of the aging lung mapped by single cell transcriptomics and deep tissue proteomics. Nat. Commun..

[83] Kjell J., Fischer-Sternjak J., Thompson A.J., Friess C., Sticco M.J., Salinas F. (2020). Defining the adult neural stem cell niche proteome identifies key regulators of adult nNeurogenesis. Cell Stem Cell.

[84] Barrett A.S., Wither M.J., Hill R.C., Dzieciatkowska M., D’Alessandro A., Reisz J.A. (2017). Hydroxylamine chemical digestion for insoluble extracellular matrix characterization. J. Proteome Res..

[85] Goddard E.T., Hill R.C., Barrett A., Betts C., Guo Q., Maller O. (2016). Quantitative extracellular matrix proteomics to study mammary and liver tissue microenvironments. Int. J. Biochem. Cell Biol..

[86] McCabe M.C., Schmitt L.R., Hill R.C., Dzieciatkowska M., Maslanka M., Daamen W.F. (2021). Evaluation and refinement of sample preparation methods for extracellular matrix proteome coverage. Mol. Cell Proteomics.

[87] Krasny L., Huang P.H. (2021). Advances in the proteomic profiling of the matrisome and adhesome. Expert Rev. Proteomics.

[88] Dussoyer M., Page A., Delolme F., Rousselle P., Nyström A., Moali C. (2021). Comparison of extracellular matrix enrichment protocols for the improved characterization of the skin matrisome by mass spectrometry. J. Proteomics.

[89] Ouni E., Ruys S.P.D., Dolmans M.M., Herinckx G., Vertommen D., Amorim C.A. (2020). Divide-and-Conquer matrisome protein (DC-MaP) strategy: an MS-friendly approach to proteomic matrisome characterization. Int. J. Mol. Sci..

[90] Knott S.J., Brown K.A., Josyer H., Carr A., Inman D., Jin S. (2020). Photocleavable surfactant-enabled extracellular matrix proteomics. Anal. Chem..

[91] Stadlmann J., Hoi D.M., Taubenschmid J., Mechtler K., Penninger J.M. (2018). Analysis of PNGase F-resistant N-glycopeptides using SugarQb for proteome discoverer 2.1 reveals cryptic substrate specificities. Proteomics.

[92] Krasny L., Huang P H. (2021). Data-independent acquisition mass spectrometry (DIA-MS) for proteomic applications in oncology. Mol. Omics.

[93] Krasny L., Bland P., Kogata N., Wai P., Howard B.A., Natrajan R.C. (2018). SWATH mass spectrometry as a tool for quantitative profiling of the matrisome. J. Proteomics.

[94] Li J., Ma J., Zhang Q., Gong H., Gao D., Wang Y. (2022). Spatially resolved proteomic map shows that extracellular matrix regulates epidermal growth. Nat. Commun..

[95] Bons J., Pan D., Shah S., Bai R., Chen-Tanyolac C., Wang X. (2022). Data-independent acquisition and quantification of extracellular matrix from human lung in chronic inflammation-associated carcinomas. Proteomics.

[96] Basak T., Vega-Montoto L., Zimmerman L.J., Tabb D.L., Hudson B.G., Vanacore R.M. (2016). Comprehensive characterization of glycosylation and hydroxylation of basement membrane collagen IV by high-resolution mass spectrometry. J. Proteome Res..

[97] Gocheva V., Naba A., Bhutkar A., Guardia T., Miller K.M., Li C.M.C. (2017). Quantitative proteomics identify Tenascin-C as a promoter of lung cancer progression and contributor to a signature prognostic of patient survival. Proc. Natl. Acad. Sci. U. S. A..

[98] Dengjel J., Bruckner-Tuderman L., Nyström A. (2020). Skin proteomics – analysis of the extracellular matrix in health and disease. Expert Rev. Proteomics.

[99] Barallobre-Barreiro J., Lynch M., Yin X., Mayr M. (2016). Systems biology-opportunities and challenges: the application of proteomics to study the cardiovascular extracellular matrix. Cardiovasc. Res..

[100] Lindsey M.L., Jung M., Hall M.E., DeLeon-Pennell K.Y. (2018). Proteomic analysis of the cardiac extracellular matrix: clinical research applications. Expert Rev. Proteomics.

[101] Arteel G.E., Naba A. (2020). The liver matrisome – looking beyond collagens. JHEP Rep..

[102] Dolin C.E., Arteel G.E. (2020). The Matrisome, inflammation, and liver disease. Semin. Liver Dis..

[103] Burgstaller G., Oehrle B., Gerckens M., White E.S., Schiller H.B., Eickelberg O. (2017). The instructive extracellular matrix of the lung: basic composition and alterations in chronic lung disease. Eur. Respir. J..

[104] Downs M., Zaia J., Sethi M.K. (2022). Mass spectrometry methods for analysis of extracellular matrix components in neurological diseases. Mass Spectrom. Rev..

[105] Kjell J., Götz M. (2020). Filling the gaps - a call for comprehensive analysis of extracellular matrix of the glial scar in region- and injury-specific contexts. Front. Cell Neurosci..

[106] Socovich A.M., Naba A. (2019). The cancer matrisome: from comprehensive characterization to biomarker discovery. Semin. Cell Dev. Biol..

[107] Naba A., Clauser K.R., Lamar J.M., Carr S.A., Hynes R.O. (2014). Extracellular matrix signatures of human mammary carcinoma identify novel metastasis promoters. eLife.

[108] Barry-Hamilton V., Spangler R., Marshall D., McCauley S., Rodriguez H.M., Oyasu M. (2010). Allosteric inhibition of lysyl oxidase–like-2 impedes the development of a pathologic microenvironment. Nat. Med..

[109] Padua D., Zhang X.H.F., Wang Q., Nadal C., Gerald W.L., Gomis R.R. (2008). TGFbeta primes breast tumors for lung metastasis seeding through angiopoietin-like 4. Cell.

[110] Hynes R.O., Naba A., Clauser K., Carr S.A., Tanabe K. (2018).

[111] Hebert J.D., Myers S.A., Naba A., Abbruzzese G., Lamar J.M., Carr S.A. (2020). Proteomic profiling of the ECM of xenograft breast cancer metastases in different organs reveals distinct metastatic niches. Cancer Res..

[112] Ray A., Provenzano P.P. (2021). Aligned forces: origins and mechanisms of cancer dissemination guided by extracellular matrix architecture. Curr. Opin. Cell Biol..

[113] Provenzano P.P., Eliceiri K.W., Campbell J.M., Inman D.R., White J.G., Keely P.J. (2006). Collagen reorganization at the tumor-stromal interface facilitates local invasion. BMC Med..

[114] Conklin M.W., Eickhoff J.C., Riching K.M., Pehlke C.A., Eliceiri K.W., Provenzano P.P. (2011). Aligned collagen is a prognostic signature for survival in human breast carcinoma. Am. J. Pathol..

[115] Tomko L.A., Hill R.C., Barrett A., Szulczewski J.M., Conklin M.W., Eliceiri K.W. (2018). Targeted matrisome analysis identifies thrombospondin-2 and tenascin-C in aligned collagen stroma from invasive breast carcinoma. Sci. Rep..

[116] Northey J.J., Barrett A.S., Acerbi I., Hayward M.K., Talamantes S., Dean I.S. (2020). Stiff stroma increases breast cancer risk by inducing the oncogene ZNF217. J. Clin. Invest..

[117] Papanicolaou M., Parker A.L., Yam M., Filipe E.C., Wu S.Z., Chitty J.L. (2022). Temporal profiling of the breast tumour microenvironment reveals collagen XII as a driver of metastasis. Nat. Commun..

[118] (2022). Obesity and Cancer Fact Sheet.

[119] Seo B.R., Bhardwaj P., Choi S., Gonzalez J., Andresen Eguiluz R.C., Wang K. (2015). Obesity-dependent changes in interstitial ECM mechanics promote breast tumorigenesis. Sci. Transl. Med..

[120] Wishart A.L., Conner S.J., Guarin J.R., Fatherree J.P., Peng Y., McGinn R.A. (2020). Decellularized extracellular matrix scaffolds identify full-length collagen VI as a driver of breast cancer cell invasion in obesity and metastasis. Sci. Adv..

[121] Mayorca-Guiliani A.E., Madsen C.D., Cox T.R., Horton E.R., Venning F.A., Erler J.T. (2017). ISDoT: *in situ* decellularization of tissues for high-resolution imaging and proteomic analysis of native extracellular matrix. Nat. Med..

[122] Fatherree J.P., Guarin J.R., McGinn R.A., Naber S.P., Oudin M.J. (2022). Chemotherapy-induced collagen IV drives cancer cell motility through activation of src and focal adhesion kinase. Cancer Res..

[123] Siegel R.L., Miller K.D., Fuchs H.E., Jemal A. (2022). Cancer statistics, 2022. CA. A Cancer J. Clin..

[124] (2022). Cancer Facts & Figures 2022.

[125] Orth M., Metzger P., Gerum S., Mayerle J., Schneider G., Belka C. (2019). Pancreatic ductal adenocarcinoma: Biological hallmarks, current status, and future perspectives of combined modality treatment approaches. Radiat. Oncol..

[126] Ray A., Callaway M.K., Rodríguez-Merced N.J., Crampton A.L., Carlson M., Emme K.B. (2022). Stromal architecture directs early dissemination in Pancreatic Ductal Adenocarcinoma. JCI Insight.

[127] Barrett A.S., Maller O., Pickup M.W., Weaver V.M., Hansen K.C. (2018). Compartment resolved proteomics reveals a dynamic matrisome in a biomechanically driven model of pancreatic ductal adenocarcinoma. J. Immunol. Regenerative Med..

[128] Tian C., Clauser K.R., Öhlund D., Rickelt S., Huang Y., Gupta M. (2019). Proteomic analyses of ECM during pancreatic ductal adenocarcinoma progression reveal different contributions by tumor and stromal cells. Proc. Natl. Acad. Sci. U. S. A..

[129] Tian C., Öhlund D., Rickelt S., Lidström T., Huang Y., Hao L. (2020). Cancer cell-derived matrisome proteins promote metastasis in pancreatic ductal adenocarcinoma. Cancer Res..

[130] Carmeliet P., Jain R.K. (2000). Angiogenesis in cancer and other diseases. Nature.

[131] Neve A., Cantatore F.P., Maruotti N., Corrado A., Ribatti D. (2014). Extracellular matrix modulates angiogenesis in physiological and pathological conditions. Biomed. Res. Int..

[132] Hanahan D. (1985). Heritable formation of pancreatic beta-cell tumours in transgenic mice expressing recombinant insulin/simian virus 40 oncogenes. Nature.

[133] Naba A., Clauser K.R., Mani D.R., Carr S.A., Hynes R.O. (2017). Quantitative proteomic profiling of the extracellular matrix of pancreatic islets during the angiogenic switch and insulinoma progression. Sci. Rep..

[134] Risson E., Nobre A.R., Maguer-Satta V., Aguirre-Ghiso J.A. (2020). The current paradigm and challenges ahead for the dormancy of disseminated tumor cells. Nat. Cancer.

[135] Linde N., Fluegen G., Aguirre-Ghiso J.A. (2016). The relationship between dormant cancer cells and their microenvironment. Adv. Cancer Res..

[136] Di Martino J.S., Akhter T., Bravo-Cordero J.J. (2021). Remodeling the ECM: implications for metastasis and tumor dormancy. Cancers (Basel).

[137] Di Martino J.S., Nobre A.R., Mondal C., Taha I., Farias E.F., Fertig E.J. (2022). A tumor-derived type III collagen-rich ECM niche regulates tumor cell dormancy. Nat. Cancer.

[138] Trombetta-Lima M., Rosa-Fernandes L., Angeli C.B., Moretti I.F., Franco Y.M., Mousessian A.S. (2021). Extracellular matrix proteome remodeling in human glioblastoma and medulloblastoma. J. Proteome Res..

[139] Sethi M.K., Downs M., Shao C., Hackett W.E., Phillips J.J., Zaia J. (2022). In-depth matrisome and glycoproteomic analysis of human brain glioblastoma *versus* control tissue. Mol. Cell Proteomics.

[140] Aebersold R., Agar J.N., Amster I.J., Baker M.S., Bertozzi C.R., Boja E.S. (2018). How many human proteoforms are there?. Nat. Chem. Biol..

[141] Burnum-Johnson K.E., Conrads T.P., Drake R.R., Herr A.E., Iyengar R., Kelly R.T. (2022). New views of old proteins: clarifying the enigmatic proteome. Mol. Cell Proteomics.

[142] Smith L.M., Agar J.N., Chamot-Rooke J., Danis P.O., Ge Y., Loo J.A. (2021). The human proteoform project: defining the human proteome. Sci. Adv..

[143] Astrof S., Crowley D., George E.L., Fukuda T., Sekiguchi K., Hanahan D. (2004). Direct test of potential roles of EIIIA and EIIIB alternatively spliced segments of fibronectin in physiological and tumor angiogenesis. Mol. Cell. Biol..

[144] Frey K., Fiechter M., Schwager K., Belloni B., Barysch M.J., Neri D. (2011). Different patterns of fibronectin and tenascin-C splice variants expression in primary and metastatic melanoma lesions. Exp. Dermatol..

[145] Rekad Z., Izzi V., Lamba R., Ciais D., Van Obberghen-Schilling E. (2022). The alternative matrisome: alternative splicing of ECM proteins in development, homeostasis and tumor progression. Matrix Biol..

[146] Santamaria-Martínez A., Huelsken J. (2013). The niche under siege: novel targets for metastasis therapy. J. Intern. Med..

[147] Shao X., Gomez C.D., Kapoor N., Considine J.M., Grams C., Gao Y.T. (2023). MatrisomeDB 2.0: 2023 updates to the ECM-protein knowledge database. Nucl. Acids Res..

[148] Izzi V., Davis M.N., Naba A. (2020). Pan-cancer analysis of the genomic alterations and mutations of the matrisome. Cancers.

[149] Trinh A., Gil Del Alcazar C.R., Shukla S.A., Chin K., Chang Y.H., Thibault G. (2021). Genomic alterations during the in situ to invasive ductal breast carcinoma transition shaped by the immune system. Mol. Cancer Res..

[150] Reid S.E., Zanivan S. (2017). Tumor stiffness extends its grip on the metastatic microenvironment. Mol. Cell Oncol..

[151] Stefanelli V.L., Choudhury S., Hu P., Liu Y., Schwenzer A., Yeh C.R. (2019). Citrullination of fibronectin alters integrin clustering and focal adhesion stability promoting stromal cell invasion. Matrix Biol..

[152] Vega M.E., Kastberger B., Wehrle-Haller B., Schwarzbauer J.E. (2020). Stimulation of fibronectin matrix assembly by lysine acetylation. Cells.

[153] Tagliabracci V.S., Engel J.L., Wen J., Wiley S.E., Worby C.A., Kinch L.N. (2012). Secreted kinase phosphorylates extracellular proteins that regulate biomineralization. Science.

[154] Tagliabracci V.S., Wiley S.E., Guo X., Kinch L.N., Durrant E., Wen J. (2015). A single kinase generates the majority of the secreted phosphoproteome. Cell.

[155] Bordoli M.R., Yum J., Breitkopf S.B., Thon J.N., Italiano J.E., Xiao J. (2014). A secreted tyrosine kinase acts in the extracellular environment. Cell.

[156] Maddala R., Skiba N.P., Rao P.V. (2017). Vertebrate lonesome kinase regulated extracellular matrix protein phosphorylation, cell shape, and adhesion in trabecular meshwork cells. J. Cell Physiol..

[157] Leutert M., Entwisle S.W., Villén J. (2021). Decoding post-translational modification crosstalk with proteomics. Mol. Cell Proteomics.

[158] Merl-Pham J., Basak T., Knüppel L., Ramanujam D., Athanason M., Behr J. (2019). Quantitative proteomic profiling of extracellular matrix and site-specific collagen post-translational modifications in an *in vitro* model of lung fibrosis. Matrix Biol. Plus.

[159] Apte S.S., Parks W.C. (2015). Metalloproteinases: a parade of functions in matrix biology and an outlook for the future. Matrix Biol..

[160] Parks W.C., Mecham R. (2011).

[161] Seals D.F., Courtneidge S.A. (2003). The ADAMs family of metalloproteases: multidomain proteins with multiple functions. Genes Dev..

[162] Mead T.J., Apte S.S. (2018). ADAMTS proteins in human disorders. Matrix Biol..

[163] Fonović M., Turk B. (2014). Cysteine cathepsins and extracellular matrix degradation. Biochim. Biophys. Acta (Bba) - Gen. Subjects.

[164] Ricard-Blum S., Salza R. (2014). Matricryptins and matrikines: biologically active fragments of the extracellular matrix. Exp. Dermatol..

[165] Ricard-Blum S., Vallet S.D. (2019). Fragments generated upon extracellular matrix remodeling: biological regulators and potential drugs. Matrix Biol..

[166] Ricard-Blum S., Vallet S.D. (2016). Proteases decode the extracellular matrix cryptome. Biochimie.

[167] Ricard-Blum S., Ballut L. (2011). Matricryptins derived from collagens and proteoglycans. Front. Biosci. (Landmark Ed..

[168] Kleifeld O., Doucet A., auf dem Keller U., Prudova A., Schilling O., Kainthan R.K. (2010). Isotopic labeling of terminal amines in complex samples identifies protein N-termini and protease cleavage products. Nat. Biotechnol..

[169] Eckhard U., Huesgen P.F., Schilling O., Bellac C.L., Butler G.S., Cox J.H. (2016). Active site specificity profiling of the matrix metalloproteinase family: proteomic identification of 4300 cleavage sites by nine MMPs explored with structural and synthetic peptide cleavage analyses. Matrix Biol..

[170] Madzharova E., Sabino F., Auf dem Keller U. (2019). Exploring extracellular matrix degradomes by TMT-TAILS N-Terminomics. Met. Mol. Biol..

[171] Bhutada S., Li L., Willard B., Muschler G., Piuzzi N., Apte S.S. (2022). Forward and reverse degradomics defines the proteolytic landscape of human knee osteoarthritic cartilage and the role of the serine protease HtrA1. Osteoarthritis Cartilage.

[172] Schlage P., Kockmann T., Sabino F., Kizhakkedathu J.N. (2015). Auf dem keller U. Matrix metalloproteinase 10 degradomics in keratinocytes and epidermal tissue identifies bioactive substrates with pleiotropic functions. Mol. Cell Proteomics.

[173] Tam V., Chen P., Yee A., Solis N., Klein T., Kudelko M. (2020). DIPPER, a spatiotemporal proteomics atlas of human intervertebral discs for exploring ageing and degeneration dynamics. Elife.

[174] Sabino F., Hermes O., Egli F.E., Kockmann T., Schlage P., Croizat P. (2015). *In vivo* assessment of protease dynamics in cutaneous wound healing by degradomics analysis of porcine wound exudates. Mol. Cell Proteomics.

[175] Swingler T.E., Waters J.G., Davidson R.K., Pennington C.J., Puente X.S., Darrah C. (2009). Degradome expression profiling in human articular cartilage. Arthritis Res. Ther..

[176] Iozzo R.V., Schaefer L. (2015). Proteoglycan form and function: a comprehensive nomenclature of proteoglycans. Matrix Biol..

[177] Rudd P.M., Karlsson N.G., Khoo K.H., Thaysen-Andersen M., Wells L., Packer N.H., Varki A., Cummings R.D., Esko J.D., Stanley P., Hart G.W., Aebi M. (2022). Essentials of Glycobiology.

[178] Ruhaak L.R., Xu G., Li Q., Goonatilleke E., Lebrilla C.B. (2018). Mass spectrometry approaches to glycomic and glycoproteomic analyses. Chem. Rev..

[179] Palaniappan K.K., Bertozzi C.R. (2016). Chemical glycoproteomics. Chem. Rev..

[180] Barallobre-Barreiro J., Baig F., Fava M., Yin X., Mayr M. (2017). Glycoproteomics of the extracellular matrix: a method for intact glycopeptide analysis using mass spectrometry. J. Vis. Exp..

[181] Eckersley A., Ozols M., Chen P., Tam V., Hoyland J.A., Trafford A. (2021). Peptide location fingerprinting reveals tissue region-specific differences in protein structures in an ageing human organ. Int. J. Mol. Sci..

[182] Eckersley A., Ozols M., O’Cualain R., Keevill E.J., Foster A., Pilkington S. (2020). Proteomic fingerprints of damage in extracellular matrix assemblies. Matrix Biol. Plus.

[183] Ozols M., Eckersley A., Mellody K.T., Mallikarjun V., Warwood S., O’Cualain R. (2021). Peptide location fingerprinting reveals modification-associated biomarker candidates of ageing in human tissue proteomes. Aging Cell.

[184] Eckersley A., Ozols M., Chen P., Tam V., Ward L.J., Hoyland J.A. (2022). Peptide location fingerprinting identifies species- and tissue-conserved structural remodelling of proteins as a consequence of ageing and disease. Matrix Biol..

[185] Pratt J.M., Simpson D.M., Doherty M.K., Rivers J., Gaskell S.J., Beynon R.J. (2006). Multiplexed absolute quantification for proteomics using concatenated signature peptides encoded by QconCAT genes. Nat. Protoc..

[186] Johnson J., Harman V.M., Franco C., Emmott E., Rockliffe N., Sun Y. (2021). Construction of à la carte QconCAT protein standards for multiplexed quantification of user-specified target proteins. BMC Biol..

[187] Carr S.A., Abbatiello S.E., Ackermann B.L., Borchers C., Domon B., Deutsch E.W. (2014). Targeted peptide measurements in biology and medicine: best practices for mass spectrometry-based assay development using a fit-for-purpose approach. Mol. Cell Proteomics.

[188] Whiteaker J.R., Halusa G.N., Hoofnagle A.N., Sharma V., MacLean B., Yan P. (2016). Using the CPTAC assay portal to identify and implement highly characterized targeted proteomics assays. Met. Mol. Biol..

[189] Whiteaker J.R., Halusa G.N., Hoofnagle A.N., Sharma V., MacLean B., Yan P. (2014). CPTAC assay portal: a repository of targeted proteomic assays. Nat. Met..

[190] Bingham G.C., Lee F., Naba A., Barker T.H. (2020). Spatial-omics: novel approaches to probe cell heterogeneity and extracellular matrix biology. Matrix Biol..

[191] Morais M.R.P.T., Tian P., Lawless C., Murtuza-Baker S., Hopkinson L., Woods S. (2022). Kidney organoids recapitulate human basement membrane assembly in health and disease. elife.

[192] Paunas F.T.I., Finne K., Leh S., Osman T.A.H., Marti H.P., Berven F. (2019). Characterization of glomerular extracellular matrix in IgA nephropathy by proteomic analysis of laser-captured microdissected glomeruli. BMC Nephrol..

[193] Piehowski P.D., Zhu Y., Bramer L.M., Stratton K.G., Zhao R., Orton D.J. (2020). Automated mass spectrometry imaging of over 2000 proteins from tissue sections at 100-μm spatial resolution. Nat. Commun..

[194] Uhlén M., Fagerberg L., Hallström B.M., Lindskog C., Oksvold P., Mardinoglu A. (2015). Tissue-based map of the human proteome. Science.

[195] HuBMAP Consortium (2019). The human body at cellular resolution: the NIH human biomolecular atlas program. Nature.

[196] Wilkinson M.D., Dumontier M., Aalbersberg IjJ., Appleton G., Axton M., Baak A. (2016). The FAIR Guiding Principles for scientific data management and stewardship. Sci. Data.

[197] Naba A., Ricard-Blum S., Ricard-Blum S. (2020). Extracellular Matrix Omics [Internet].

[198] Shao X., Taha I.N., Clauser K.R., Gao Y.T., Naba A. (2020). MatrisomeDB: the ECM-protein knowledge database. Nucl. Acids Res..

[199] Desiere F., Deutsch E.W., King N.L., Nesvizhskii A.I., Mallick P., Eng J. (2006). The PeptideAtlas project. Nucl. Acids Res..

[200] Fortelny N., Yang S., Pavlidis P., Lange P.F., Overall C.M. (2015). Proteome TopFIND 3.0 with TopFINDer and PathFINDer: database and analysis tools for the association of protein termini to pre- and post-translational events. Nucl. Acids Res..

[201] Richards A.L., Eckhardt M., Krogan N.J. (2021). Mass spectrometry-based protein–protein interaction networks for the study of human diseases. Mol. Syst. Biol..

[202] Clerc O., Deniaud M., Vallet S.D., Naba A., Rivet A., Perez S. (2019). MatrixDB: integration of new data with a focus on glycosaminoglycan interactions. Nucl. Acids Res..

[203] Kontio J., Soñora V.R., Pesola V., Lamba R., Dittmann A., Navarro A.D. (2022). Analysis of extracellular matrix network dynamics in cancer using the MatriNet database. Matrix Biol..

[204] Jayadev R., Morais M.R.P.T., Ellingford J.M., Srinivasan S., Naylor R.W., Lawless C. (2022). A basement membrane discovery pipeline uncovers network complexity, regulators, and human disease associations. Sci. Adv..

[205] Karamanos N.K., Piperigkou Z., Passi A., Götte M., Rousselle P., Vlodavsky I. (2021). Extracellular matrix-based cancer targeting. Trends Mol. Med..

[206] Huang J., Zhang L., Wan D., Zhou L., Zheng S., Lin S. (2021). Extracellular matrix and its therapeutic potential for cancer treatment. Sig Transduct Target Ther..

[207] Naba A., Clauser K.R., Ding H., Whittaker C.A., Carr S.A., Hynes R.O. (2016). The extracellular matrix: tools and insights for the “omics” era. Matrix Biol..

